# Supplementary material for: Urinary incontinence among pregnant women in Southern Brazil: A population-based cross-sectional survey
Source: PLoS One. 2020 Jun 8;15(6):e0234338. doi: 10.1371/journal.pone.0234338 (PMC7279605; doi:10.1371/journal.pone.0234338)
Supplement: Attachment — Submitted filename: Response to reviewers.docx [file pone.0234338.s001.docx]

**Response to reviewer**

**REVIEWER #2: DEAR AUTHOR!**

**General:**

Why did you not separate the conditions stress urinary incontinence and urgency urinary incontinence, which are quite different in etiology?

**Answer**: we understand that the separation of the etiology of urinary incontinence is important at the time of treatment, and we aim to measure the prevalence of this condition in the studied population. Notwithstanding this, a brief citation of the types of urinary incontinence in this research was made in the Result section.

**Abstract:**

- UI is a frequent pathology = UI is a frequent condition. **Answer**: done. Line 23

- Measure the prevalence = estimate the prevalence. **Answer**: done. Line 25

**Background:**

“…and increases with age” =”…and the prevalence increases with advancing age”- This is a journal read mainly by health care professionals, it is therefore unnecessary to explain that UI may be experienced by both sexes.

**Answer**: The new wording of this excerpt looks like this: “Urinary incontinence (UI) is defined as any involuntary loss of urine and the prevalence increases with advancing age ^(1)^. Among women, its occurrence ranges from 40% to 60% among women, and from 10% to 20% among men ^(2)^.” Line 56 – 58.

- What is essential, however, is that you use the background section to focus on why it is important to examine UI in your population, when similar studies have been performed in other populations. Are you f.i. suspecting that women in Rio Grande would be especially prone to UI due to (I am just guessing) heavy rural work or young age at first delivery?

**Answer**: I do not hypothesize that women in the rural area are more likely to suffer UI than the others. Less than 5% of births originated from rural areas, and no questions that sought to better characterize this problem were formulated. Moreover, physical exertion has not been investigated in detail.

- Rewrite the following, since in its present form it seems like all factors should be present in the same patient: “The probability of occurrence of UI, even after adjustment, was significantly higher among those who were older than 30 years old at current pregnancy, whose first pregnancy was before the age of 20 or after 30, who reached the end of gestation weighing 90 kg or more, who exercised regularly during pregnancy and who reported frequent urinary urgency during the gestational period.”

**Answer**: This paragraph was modified as follows:

“After adjusted analysis, the likelihood of UI occurring varied significantly as per women’s characteristics. For example, the PR for the occurrence of UI among women over 30 years of age was 2.05 (95% CI: 1.39-3.01) compared to adolescents. In two other groups of women who had their first pregnancy before the age of 20 or after the age of 30, the PR for UI was 1.36 (95% CI: 1.04-1.76) and 1.59 (95% CI: 1.01-2.51), respectively, when compared to those who became pregnant for the first time between 20 and 29 years of age. Finally, in two other groups of women, namely, those who reached 90 kg and over at the end of pregnancy and those who performed regular physical exercise and reported frequent urinary urgency, the PR was 2.49 (95% CI: 1.74-3.57), and 2.90 (95% CI: 2.10-4.00) compared to those who did not exercise and did not report urinary urgency, respectively.” Line 37 – 48.

**Methods:**

- First paragraph-shorten or remove

**Answer**: This paragraph has been removed. Line 79 – 80.

- Why cut-off at 20 weeks?

**Answer**: This is a study of mothers who had live births or stillbirths. Epidemiological studies have classified them as abortion when below 20 weeks or 500 grams, and they were not eligible for this study. Their mothers originated from a survey that evaluated pregnancy and childbirth care in the municipality.

- Power-calculation hard to follow, and must be better described: especially the following part “…ratio between exposed and unexposed/exposed of 15/85, prevalence of disease among the unexposed of 9.8% and 1.6 risk ratio..”

**Answer**: This is a standard description, where the parameters are displayed. If this seems sufficient, this text may be replaced with one that is a little more detailed. This passage:

“*Regarding the identification of associated factors, for alpha error of 0.05, beta error of 0.20, ratio between exposed and unexposed/exposed of 15/85, prevalence of disease among the unexposed of 9.8% and 1.6 risk ratio, at least 2,680 puerperae should be included in the study. “*

Was replaced by this one:

“Regarding the identification of associated factors, the parameters used were as follows: significance level of 95% (or alpha error of 0.05), power of 80% (or beta error of 0.20), a proportion of one exposed for about six not exposed to UI (that is, 15/85), UI prevalence of 9.8% among women not exposed to the factors under study, and up to 15.7% among those exposed to these same factors, which implies a risk ratio of 1.6 (9.8 x 1.6 = 15.7%). The study should include at least 2,680 puerperae to work with these parameters.” Line 94 – 100.

- eventual losses = eventual lost to follow-up/ lost data or deaths?

**Answer**: The losses refer to women who were not submitted to the questionnaire, either because they refused to participate in the study or because they left the hospital before the mandatory minimum 48-hour stay. As this is a cross-sectional study, that is, with a single approach, follow-up losses could not be incurred. No deaths within 48 hours after delivery were recorded among all those who took part in this study.

- Remove repeated statements like, like p.4 “…the following question: "During this gestation, did you ever lose urine unintentionally?" A positive response would mean puerperae had UI during this gestational period.”

**Answer**: done, line 102 – 104.

- Condense the text from “Three interviewers collected data….” to “…was performed using Stata statistical package version 12.0. “

**Answer**: done. Line 108 – 111.

- When were the 10% of the interviews retaken? Did you consider recall bias? What happened when the data were inconsistent, which questionnaire was used- the first or the second?

**Answer**: These partial interviews were retaken within two weeks after delivery. Given the short time between the two moments and the type of question asked, such as type of delivery, we believe that there was no recall bias. Any inconsistency would validate and use the information provided on the second interview, which was very rarely the case.

- Explain the advantage of using Poisson regression models in non-longitudinal data instead of regular linear or logistic regression?

**Answer**: We used logistic regression because we work with a binary outcome of high prevalence (14.7%), with irregular distribution of cases and with a measure of effect (prevalence ratio) that is easily understood by non-specialists (odds ratio). Furthermore, this procedure avoids overestimating the measure of effect, which occurred when using logistic regression with the odds ratio as a measure of effect. Our procedure builds on these references: Barros AJ, Hirakata VN. Alternatives for logistic regression in cross-sectional studies: an empirical comparison of models that directly estimate the prevalence ratio. BMC Med Res Methodol. 2003; 3-21 & Kirkwood BR, Sterne JAC. Essentials of medical statistics. 2nd ed. London: Blackwell Scientific Publications; 2003.

- Remove inadequate racial expressions like skin color, white, brown, black etc. (also in results section and tables)

**Answer**: We do not agree that the terms used are inappropriate. We only measured subjectively the skin pigmentation’s tone. Those who were not white or black were classified as brown. We saw nothing more than that. No other characteristics that would allow us to discourse, for example, on ethnicity or race, were evaluated. This is very difficult in Latin America, especially in Brazil, where the European, Caucasian, mixed with the native Indian and the African. It would be much easier in Europe, or even in North America, but not in Brazil. Furthermore, several publications in Brazil, but also Europe and the United States adopt these terms. For these reasons, the terms we employed seem the most appropriate, but if the reviewer or the editor has a different classification, and manages to convince us that they are more appropriate than ours, we would more than happy to change them.

**Results:**

- Incredible that more than 99% participated voluntarily in a study, how was this possible?

**Answer**: It is true. There are at least two reasons for this: 1) this study is conducted entirely on the premises of the only two local hospitals and this makes it much easier to reach mothers at the time that is most suitable for them and 2) it is the fourth study carried out by our team. This led to establishing a relationship of trust not only with the management of these hospitals, and health professionals, but also with mothers, because countless actions had already taken place in the municipality based on the results of these surveys, and they end up knowing about it.

- Do not repeat extensively in text what is presented in the tables

**Answer**: done. Line 152 – 156.

**Discussion:**

- Modify strong expressions such as “this is due to the loss of….”, rather use “this may be due to..” or similar.- Great age= old age/ advanced age

**Answer**: done. Line 189.

**Conclusions:**

- Avoid the expression causally associated, since to detect causal effects from a cross-sectional study would be very hard.

**Answer**: done. Line 258.
