# Supplementary material for: Urinary incontinence among pregnant women in Southern Brazil: A population-based cross-sectional survey
Source: PLoS One. 2020 Jun 8;15(6):e0234338. doi: 10.1371/journal.pone.0234338 (PMC7279605; doi:10.1371/journal.pone.0234338)
Supplement: Attachment — Submitted filename: Response to reviewers II.docx [file pone.0234338.s002.docx]

**Response to reviewer**

Dear Reviewer #2

Here are your comments and our answers, thank you for considering our efforts for improving this paper:

- You do not have to over-explain the power calculation in a statistical sense, however I am asking you to explain or reference your assumptions. Where did the ratio of unexposed/exposed to UI come from? I suppose the power calculation was performed before you started the study and not performed post-hoc?

**Answer**: We made new adjustments to not over-explain the power calculation. However, for answering the question, the calculation of the sample size, we used data published in other studies and a pilot study carried out before the beginning of data collection. The formula used to calculate the sample parameters was this: A/(B+C*E), where: A=Prevalence of outcome; B=Proportion of non-exposed; C=Proportion of exposed; D=Prevalence of outcome among non-exposed; E=Prevalence ratio (Dean AG, Dean JA, Coulombier D, Brendel KA, Smith DC, Burton AH, Dicker RC, Sulliven K, Fagan RF, Arner TG. Epi-Info, Version 6.02: A Word Processing, Database, and Statistics Program for Epidemiology on Microcomputers. Atlanta (GA): Centers of Disease Control and Prevention; 1994). (Line 93 -98)

- The expression “losses” should be briefly explained: for instance: ..losses, including women who did not want to participate in the study or who left the hospital before being invited to participate….

**Answer**: Done. Line 95 -96.

- I still find the entity “skin color” problematic. Since the Brazilian population is ethnically mixed, the genetic importance of skin color is dubious. Because skin color does not have any relevance for your results, and in order to avoid offending readers, I would recommend the removal of this entity. However, I leave the decision to the editor.

**Answer**: We reiterate that we do not evaluate ethnicity, but only the color of the skin, for the reasons already explained in a previous message. In Brazil, saying that someone has white, brown or black skin color is not offensive and has been widely used in scientific articles. I kindly ask you to look for articles by Victora CG, Barros FC and other Brazilian authors, where you can see the wide use of this name. If this does not convince the reviewer, then we leave it to the editor to decide.
